# Learning with a digital escape room game: before or after instruction?

**DOI:** 10.1186/s41039-022-00187-x

**Published:** 2022-03-15

**Authors:** Josef Buchner, Martina Rüter, Michael Kerres

**Affiliations:** 1 Learning Lab, University of Duisburg-Essen, Universitätstraße 2, 45141 Essen, Germany; 2 Bochum University of Applied Sciences, Am Hochschulcampus 1, 44801 Bochum, Germany

**Keywords:** Educational escape game, Escape room game, Digital escape game, Cognitive load, Productive failure, Open educational resources, Problem-solving, Instructional design

## Abstract

In this study, we investigated whether playing an escape room game after explicit instruction (instruction-first group, *N* = 20) is more effective to learn about copyright and media law than playing the game before explicit instruction (problem-solving-first group, *N* = 21). This is an important question as escape room games are complex, problem-based learning environments that can overwhelm learners and thus hinder the acquisition of knowledge and skills. According to cognitive load theory, preparing learners with explicit instruction before problem-solving activities can overcome this problem leading to better learning. However, in the productive failure paradigm problem-solving before explicit instruction is seen as more effective, especially when it comes to the application of newly acquired knowledge to solve novel problems. Based on these two theories, we conducted an experiment and found that playing the developed digital escape room game after explicit instruction was more effective for knowledge retention and domain-specific self-efficacy with at the same time lower cognitive load. However, we found no differences regarding the application of knowledge as both groups scored equally high on transfer tasks. The result is discussed considering previous productive failure studies mostly conducted in the science and engineering domain. In sum, the instruction first approach proved to be effective for both knowledge acquisition and knowledge application, leading to higher domain-specific self-efficacy and lower cognitive load. Therefore, we conclude that the implementation of escape room games after instruction is an effective instructional approach and better suited to promote learning than playing escape room games before instruction.

**Supplementary Information:**

The online version contains supplementary material available at 10.1186/s41039-022-00187-x.

## Introduction

Escape room games (ERGs) are defined as *live-action team-based games where players discover clues, solve puzzles and accomplish tasks in one or more rooms in order to accomplish a specific goal (usually escaping from the room) in a limited amount of time* (Nicholson, 2015, p. 1). These games gained popularity in recent years in the recreation and entertainment industry resulting in an increase of companies providing escape experiences in which players are immersed in a narrative-based challenge, e.g., save the world, and directly engage with an authentic game world (Nicholson, 2018; Sanchez & Plumettaz-Sieber, 2019). The combination of hands-on and mind-on activities within an authentic environment inspired teachers and instructors worldwide to adapt the concept of escaping the room for educational purposes (Veldkamp, van de Grint, et al., 2020). Educational escape games (EEG) can be defined as *an instructional method requiring learners to participate in collaborative playful activities explicitly designed for domain knowledge acquisition or skill development so that they can accomplish a specific goal (e.g., escape from a physical room or break into a box) by solving puzzles linked to unambiguous learning objectives in a limited amount of time* (Fotaris & Mastoras, 2019, p. 236). An adaptation of the traditional game play of ERG is necessary when used in education, since it is not allowed to lock students into a room and wait until they find their way out (Fotaris & Mastoras, 2019). Therefore, educators developed solutions using physical boxes or vaults in which learners have to break in with the help of both analog and digital materials. Such EEGs are described as hybrid learning spaces simulating a real ERG atmosphere (Veldkamp, Daemen, et al., 2020a, b), for example by integrating videos, QR-codes or augmented reality elements (Borrego et al., 2017; Estudante & Dietrich, 2020). More recently, also because of the global COVID-19 pandemic, fully digital EEGs are gaining interest as they allow learners to play at home (Makri et al., 2021).

The educational benefits of EEGs are many, ranging from fostering cognitive and affective learning outcomes to developing teamwork, problem-solving, communication and creativity skills as well as promoting career interest in science, technology, engineering and mathematics (STEM) professions (e.g., Fotaris & Mastoras, 2019; Sanchez & Plumettaz-Sieber, 2019; Veldkamp et al., 2021; Veldkamp, van de Grint, et al., 2020). Theoretically, researchers explain the positive effects of EEG considering the active learning paradigm and game-based learning theories (Nicholson, 2015, 2018). Thereby, learning with EEG is associated with increased learner engagement and motivation, which in turn can positively affect learning achievement. For example, Franco and DeLuca (2019) developed an EEG for doctoral students in healthcare leadership and found that the game enhanced the overall learning experience and allowed learners to not only memorize information but to apply the newly acquired knowledge (p. 40). Lopez-Pernas et al. (2019b) provide evidence for the entertaining and engaging effect of an EEG when learning programming. Students reported to like learning with the developed EEG and stated that playing helped them to foster their knowledge in the course (p. 31731). In a second study, Lopez-Pernas et al. (2019a) also present results of a pre-posttest design about the effectiveness of the EEG showing that the students improved their knowledge after playing the game with a medium to large effect size (p. 184232). However, the results were not compared to a control group. In general, research on the effectiveness of EEGs is at an early stage as published review studies reveal. In Veldkamp, van de Grint et al. (2020) three studies were found that evaluated knowledge acquisition in a pre-posttest design and only one study established a design with a control group (Cotner et al., 2018). In the review by Makri et al. (2021) four studies were identified that measured learning performance. A closer look at the studies further reveals that no study has investigated at what point in an instructional design an EEG works best. However, this is an important question, as research on games for learning has shown that they can also overwhelm learners due their explorative and problem-based nature leading to poorer learning (Mayer, 2019; Westera, 2019). For example, in Hermanns et al. (2017) students learned in pharmacology education with an EEG and rated the learning experience as engaging and a good opportunity to collaborate with others but they also expressed feelings of frustration and cognitive overload (e.g., students did not understand where to start the activity). In Veldkamp et al. (2021), both teachers and learners doubt that the used EEG for biology education can help them acquiring new knowledge in the domain. The reason given for this assessment is the unstructured nature of the escape room experience not allowing to understand the content of the lesson (p. 8). In sum, EEGs like other educational games are explorative, problem-based learning scenarios that may be fun, but do not really contribute to learning without further instructional elements. This effect can be explained by considering cognitive load theory (CLT): CLT is an instructional design theory grounded on the human cognitive architecture consisting of a sensory register, a working memory with limited capacity and a long-term memory with unlimited storage size (Sweller, 1988; Sweller et al., 1998, 2019). Most important in CLT is the limited working memory capacity. The aim of instruction is to process new information in working memory by integrating already stored knowledge and to transfer the new knowledge into long-term memory. This process works best if the working memory capacity is not overloaded. Hence, critical for effective learning is the cognitive load imposed during instruction (Sweller et al., 2019). Sweller (2020) distinguishes two types of cognitive load: Intrinsic cognitive load (ICL) and extraneous cognitive load (ECL). ICL arises due the complexity of the task and is directly relevant for learning. ECL is also known as the unproductive or unnecessary cognitive load that hinder learning caused by the instructional design, learner characteristics or aspects of the learning environment. Effective instruction reduces the ECL to foster germane processing (productive cognitive processing) resulting in more working memory capacity to handle the ICL of a learning task (Paas & van Merriënboer, 2020). As a consequence, CLT researchers argue in favor of the direct instruction approach in which explicit explanations and information is presented to the learners before solving unstructured problems. A large body of empirical research supports this claim by proofing better knowledge acquisition with direct instruction compared to unguided instructional approaches (Kirschner et al., 2006; Sweller et al., 2007).

However, other researchers argue that the achievement of learning objectives beyond knowledge acquisition, like the application of knowledge to solve new tasks (i.e., transfer performance), can also be reached by instructional approaches that provide explicit information after a problem-based learning activity (Hmelo-Silver et al., 2007; Kapur, 2008). This instructional approach is called productive failure (PF), which involves a problem-solving phase followed by an explicit instructional phase (Kapur, 2014, 2016). Several reasons can explain the positive effect of PF (Kapur, 2014, p. 1009): Problem-solving tasks before instruction can activate the prior knowledge of the learners indicating to them learning gaps that can be used to effectively improve knowledge by selecting the relevant information in the subsequent instructional phase. In addition, the cognitive load imposed through a complex problem-solving task prior to instruction can boost learners engagement and motivation, which in turn compensates for the high cognitive demand (e.g., Likourezos & Kalyuga, 2017). The advantage of PF over direct instruction for transfer performance has been demonstrated in some studies, hence it can be concluded that PF is an effective instructional approach (e.g., Kapur, 2014; Loibl et al., 2017; Sinha & Kapur, 2021). However, there are also studies that have found no benefits of PF over direct instruction for both retention and transfer performance (e.g., Halmo et al., 2020; Likourezos & Kalyuga, 2017; Nachtigall et al., 2020). Furthermore, the majority of the studies demonstrating advantages of PF over direct instruction were conducted for learning in STEM domains. A first research study investigating PF in non-STEM domains was not able to replicate its effectiveness (Nachtigall et al., 2020). However, more research is particularly needed in domains others than science and mathematics to examine the possible domain-independence of the PF approach (Kapur, 2015; Loibl et al., 2017).

We respond to this request and situate our study in the non-STEM learning domain of media and copyright law with a special focus on the creative commons (CC) licensing model. The topic of CC licensing is of great interest in teacher education and training as it is directly related to the open education movement (Otto, 2019). However, learning how to properly label and find open educational resources (OER) is complex and requires not only knowledge but also specific skills (Tlili et al., 2021). An open question here is, how learners can most effectively be supported in acquiring the necessary knowledge and skills (Otto et al., 2021). As Otto et al. (2021) point out, empirical investigation considering contemporary educational technologies, methods and theories is needed to provide evidence-based recommendations for practice on how to effectively design courses in the field of copyright and media law. In this research, we use the contemporary method of an EEG and apply CLT and PF to contribute to the evidence base on how to effectively support learners in the copyright and media law domain. The use of an EEG in the domain of copyright and media law can be justified on the basis of the literature already outlined: EEGs allow for the application of knowledge (e.g., Franco & DeLuca, 2019), a key learning objective in media law courses. However, as shown, without instructional support, learning within EEGs can be perceived as overwhelming. Therefore, it is worth investigating at what point in an instructional design learners benefit in both knowledge and skills acquisition. Additionally, we were interested in the impact of our instructional design on learners’ self-efficacy. As Otto (2021) has shown, belief in one's own abilities is central when it comes to subsequent applications of content from copyright and media law courses. CLT and PF provide a theoretical and empirical basis to investigate these issues. Consequently, we assess cognitive load to explain the results found. In sum, we explore in this research the question, whether learning with a digital ERG in the domain of copyright and media law is more effective when explicit instruction is provided before or after playing.

The remaining structure of the paper is as follows: First, we describe our methodological approach and present the hypotheses of this study. In the following, we report the results of the experiment and discuss them in a further section. The paper closes with some limitations of our study and future research directions. At the end, we provide conclusions and recommendations for practitioners how to integrate EEGs into teaching.

## Method

### Research question and hypotheses

As mentioned above, we investigate in this study the question if learning with a digital ERG is more effective when integrated in an instructional design in which explicit instruction is provided before or after playing the game. To do so, we conducted an experiment and defined three learning outcomes and a learning process variable to gain insights into the effectiveness: The first learning outcome is knowledge acquisition in the form of knowledge retention. The second learning outcome is the application of the acquired knowledge to solve new tasks representing the learners transfer performance. The third learning outcome is the perceived self-efficacy in the learning domain. Self-efficacy is defined as a person’s subjective belief to successfully perform actions in a specific domain or in general (Bandura, 1977, 1994). We focus in this research on domain-specific self-efficacy as a learning outcome. Based on the aforementioned findings on CLT and PF, it can be assumed that the instructional design influences self-efficacy. For example, more explicit instructional approaches promote knowledge acquisition to a greater extent resulting in a higher belief in one’s own ability to solve new problems (e.g., Huang, 2017). To understand the learning process, we measured cognitive load levels of the learners. Considering these variables, we test the following hypotheses in this study:

- Hypothesis 1: Playing the ERG after explicit instruction is more effective for knowledge retention than playing the game before explicit instruction.
- Hypothesis 2: Playing the ERG before explicit instruction is more effective for transfer performance than playing the game after explicit instruction.
- Hypothesis 3: Playing the ERG after explicit instruction results in higher domain-specific self-efficacy than playing the game before explicit instruction.
- Hypothesis 4: Playing the ERG after explicit instruction leads to lower cognitive load than playing the game before explicit instruction.

### Participants, context and learning domain

A total of 41 learners (14 males) participated in this study. The majority of the participants were students (21) with an age between 21 and 29 years. The other participants classified themselves as teachers (9), lecturers (3), academic staff (4), instructor in further education (1) or other (3) aged between 18 and 60. The participants were either attendees of a university course taught by the second author or persons with an interest in the learning domain and therefore participated in the experiment. As mentioned above, the learning domain was copyright and media law for teachers and lecturers with a focus on the creative commons (CC) licensing model. The aim was to learn how to label educational materials as open educational resources (OER) using the right CC license. This is an important educational goal in the training of teachers and educators to further strengthen the paradigm of open education (Otto, 2019).

### Research design

The independent variable in this study was the instructional design: In one group, participants first engaged in explicit instruction delivered through a web-based training (WBT) followed by an escape room game (ERG). In the following, we will refer to this group as the instruction first (IF) group. In the other group, participants first studied with the ERG followed by the WBT. In the following, we will refer to this group as the problem-solving first (PSF) group. All 41 participants were randomly assigned either to the IF group (*N* = 20; eight males) or the PSF group (*N* = 21; six males). The dependent variables in this study were knowledge acquisition (retention), knowledge application (transfer), domain-specific self-efficacy and cognitive load (intrinsic and extraneous cognitive load). It is important to note that in this study individual learning with the respective instructional design was implemented.

### Learning materials

#### Web-based training (WBT)

**Fig. 1:**
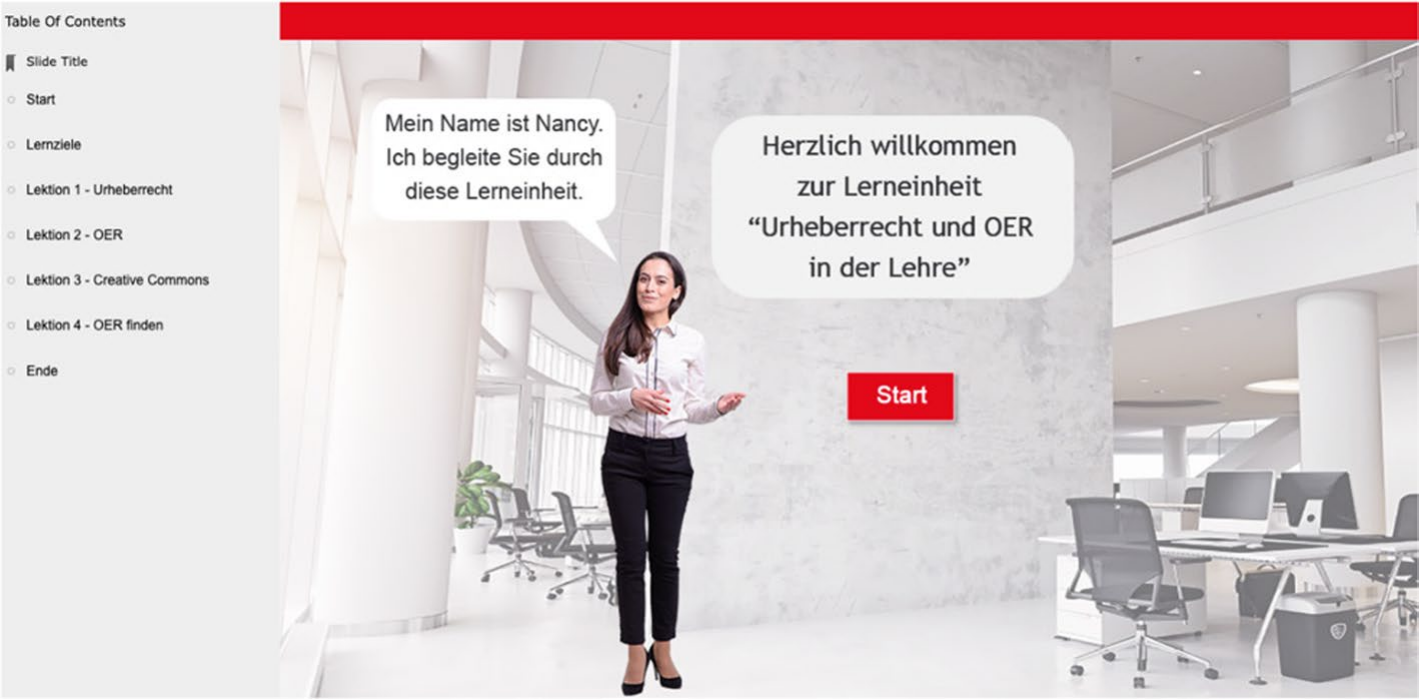
Start of the WBT with table of contents

The WBT was designed using Adobe Captivate© software (e.g., Duvall, 2014). The aim of the WBT was to deliver basic knowledge in the learning domain of copyright and media law with texts, pictures and videos (see Fig. 1). The WBT is an explicit instructional material focusing on information and content presentation allowing learners to engage with in a self-paced manner (e.g., Kerres & de Witt, 2003). The WBT used in this study was developed by the second author and is available for interested educators in the German language as an open educational resource (OER) at wbt.online-lernkurse.de.

#### Escape room game (ERG)

**Fig. 2:**
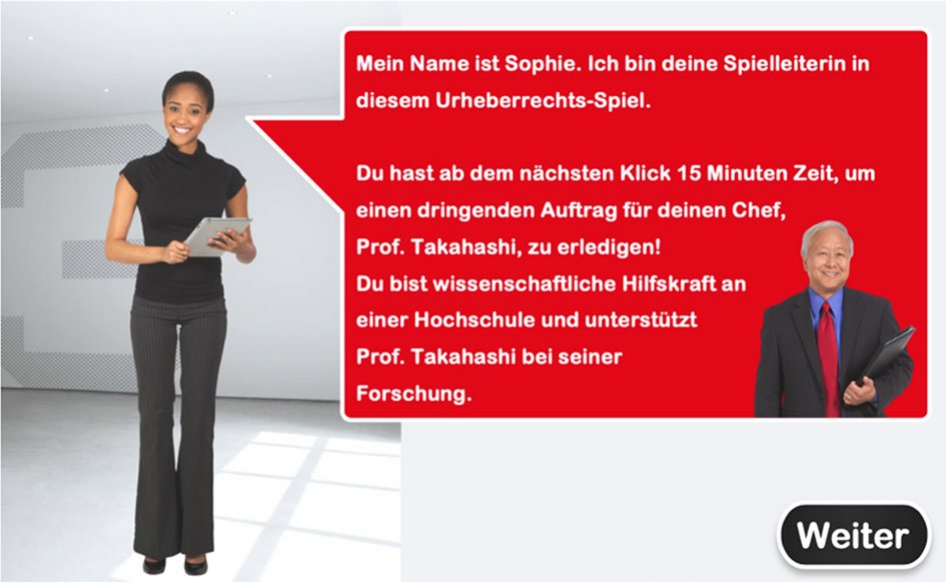
Female and male avatar that support the storyline and the player during the game

The digital ERG was developed using Articulate Storyline© software (e.g., Mitropoulou & Argyropoulos, 2020). The aim of the ERG was to practice by completing a task under time pressure. The completion time of the ERG was limited to 15 min visualized by a countdown timer. If the time runs out, the game ends and has to be started from the beginning. The ERG is designed sequentially and embedded in a frame story in which the players take on the role of a research assistant. The problem-based task is to replace an illustration in an article to be submitted by the editorial deadline (in 15 min). In the process, the players encounter various questions about copyright, the OER search and CC licensing model. A virtual game leader (female avatar, see Fig. 2) leads through the game, explaining the task at the beginning and providing motivational feedback as the game progresses. The task types were single- or multiple-choice, drag-and-drop tasks and text fields in which texts or numbers (codes) had to be entered. The single- or multiple-choice tasks are not presented in a classical list with checkboxes or radio buttons, but also by clicking on depicted objects, in order to support the game character of the ERG. The feedback texts were mainly designed to briefly explain the correct solution in each case. Also, the navigation was mainly done by clicking on objects with the mouse or by a continue button, which only appears after a question has been answered correctly. At the end of the game, the remaining time is displayed.

**Fig. 3:**
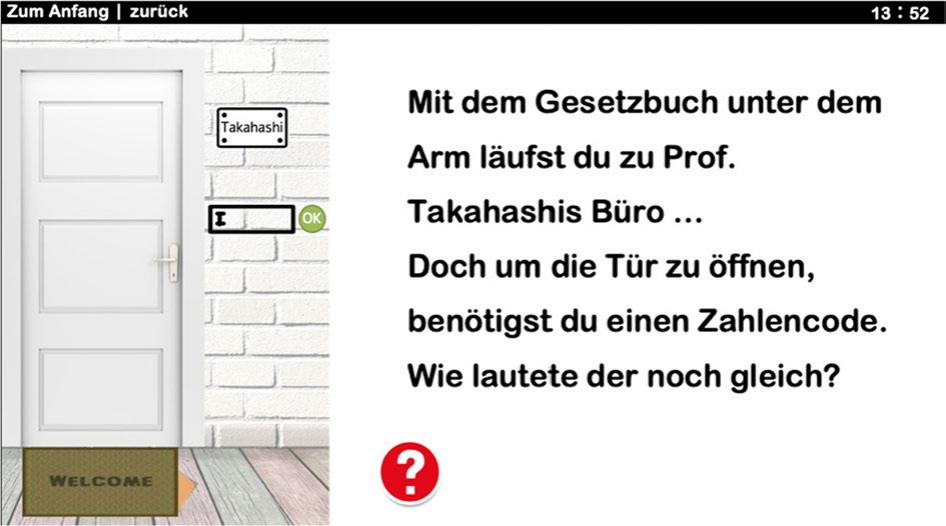
Example of the open-the-door puzzle; the red button with the question mark displays a hint when clicked; at the upper right corner is the timer

The game starts with an e-mail from Prof. Takahashi (male avatar, see Fig. 2), which contains the task to be completed. The players are first sent to the library to find out about copyright law. Then they have to enter the door code to Prof. Takahashi’s office. The numerical code results from the answer to two questions about copyright (see Fig. 3). In the further course of the game, a PC password must be entered in order to access the article to be changed. This is followed by an Internet search for OERs. At the end of the game, the players have to answer questions about CC licensing model correctly in order to quote the new illustration accurately.

The ERG used in this study was developed by the second author and is available for interested educators in the German language as an open educational resource (OER) at oer.online-lernkurse.de.

### Instruments

#### Prior knowledge

To consider participants’ prior knowledge in the learning domain of copyright and media law, we used a self-reporting scale from 1 = no prior knowledge to 7 = high prior knowledge. We do not applied a full knowledge test to avoid the problem of a possible testing effect (Johnson & Mayer, 2009).

#### Knowledge acquisition

To assess learners’ knowledge acquisition, we developed a multiple-choice questionnaire with six items (see Additional file 1: Supplementary Material) in collaboration with a media law attorney and an expert in the field of OER. For example, “How many CC licenses are included in the Creative Commons licensing model? 4, **6**, 9”. Four questions were rated as easy and rewarded with one point each if correct. The remaining two questions were rated as difficult and rewarded with two points each if correct. Only fully correct questions were rewarded with points, a total of eight points was possible to reach. The results of the multiple-choice questionnaire represent learners’ retention performance.

#### Knowledge application

To measure whether participants can apply their knowledge, we developed a transfer test with six text-based tasks (see Additional file 1: Supplementary Material). Again, the development of the transfer tasks was done in collaboration with the media law attorney and the expert in the field of OER. For example, “An image is licensed under CC BY-SA 2.0. What license do you give the image after editing (e.g., cropping)? CC-BY 2.0, **CC-BY-SA 2.0**, CC-BY-SA 2.1, CC-BY-SA-ND 4.0”*.* Three transfer tasks were rated as easy and rewarded with three points each if correct. The remaining three were rated as difficult and rewarded with four points each if correct. In sum, participants could reach 21 points in the knowledge application task representing learners’ transfer performance.

#### Domain-specific self-efficacy

To survey participants self-efficacy in the domain of copyright and media law, we adapted and supplemented the items developed by Van Acker et al. (2013). The new questionnaire consists of six items, for example, “I feel confident about copyright issues”*.* Participants answered the items on a scale from 1 = do not agree to 7 = fully agree, *Cronbachs Alpha* = 0.89.

#### Cognitive load

In order to assess participants cognitive load, we used two subscales of the instrument developed by Klepsch et al. (2017): Intrinsic cognitive load (ICL; 2 items; e.g., “This task was very complex”; *Cronbach’s Alpha* = 0.69) and extraneous cognitive load (ECL; 3 items; e.g., “The design of this task was very inconvenient for learning”; *Cronbach’s Alpha* = 0.66). Participants answered the items on a scale from 1 = do not agree to 7 = fully agree.

### Procedure

**Fig. 4:**
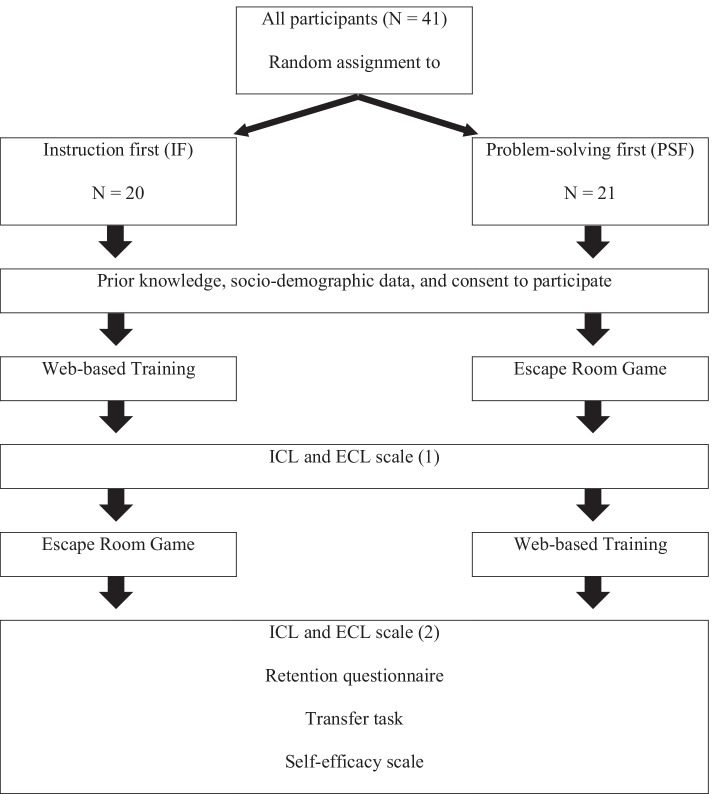
Research procedure

The study was conducted as a fully online experiment in which participants were enrolled in a *Moodle* course and automatically assigned to the instructional design of IF or PSF by the system. First, participants answered the prior knowledge rating scale, the socio-demographic questions and agreed to participate in the study. It is important to note here that all data collected were analyzed anonymously. Afterwards, participants learned according to their assignment with the WBT (IF group) or the ERG (PSF group). After completing, the learners answered the ICL and ECL scale for the first time and continued learning either with the ERG (IF group) or the WBT (PSF group). Thereafter, the participants again completed the ICL and ECL scale as well as the knowledge acquisition questionnaire, the transfer task and the self-efficacy scale (overview in Fig. 4).

## Results

For data analysis purposes, all items in all instruments were aggregated to their respective scale. The scorings of the retention test and transfer task were summed. The domain-specific self-efficacy scale was computed using mean values. The results of the two ICL and ECL scales (1 + 2) were aggregated to an overall ICL and ECL scale using mean values. In Table 1, descriptive data of all measured variables of this study are presented.

**Table 1:** Descriptive statistics of both groups for all measured variables

	IF group (*N* = 20)		PSF group (*N* = 21)	
	*M*	SD	*M*	SD
Prior knowledge	3.27	1.76	3.16	1.59
Retention	5.25	1.71	4.05	2.20
Transfer	16.25	4.46	15.95	4.86
Self-efficacy	5.30	0.98	4.66	1.29
Overall ICL	2.58	1.04	3.33	1.12
Overall ECL	1.94	0.80	2.29	0.83

Initially, we calculated a Shapiro–Wilk test to explore if the data of the dependent variables are normally distributed. The results show that, except for two scales, no normal distribution of the data can be assumed. As a consequent, we test the hypotheses of the study using non-parametric procedures like Mann–Whitney test. All calculations were performed in SPSS 27. Effect sizes are given according to Cohen (1988).

### Prior knowledge

First, we checked if the participants differ in their prior knowledge in the learning domain. As the descriptive data in Table 1 shows, in both groups learners report low and similar prior knowledge. Further, a Mann–Whitney test confirms that the participants do not differ in their prior knowledge in the learning domain (IF group *mean rank* = 21.30, PSF group *mean rank* = 20.71, *U* = 204.00, *z* = − 0.16, *p* = 0.88).

### Knowledge acquisition – retention

Descriptive data in Table 1 support the first hypothesis: The learners in the IF group (*M* = 5.25, *SD* = 1.71) performed better on the retention test than the learners in the PSF group (*M* = 4.05, *SD* = 2.20). A Mann–Whitney test shows that the difference between the groups is significant with a moderate effect size: IF group *mean rank* = 24.33, PSF group *mean rank* = 17.83, *U* = 143.50, *z* = − 1.76, *p* < 0.05 one-tailed, *d* = 0.56.

### Knowledge application – transfer

As presented in Table 1, participants in the IF group (*M* = 16.25, *SD* = 4.46) and the PSF group (*M* = 15.95, *SD* = 4.86) performed similar in the transfer task after learning with the respective instructional design. Consequently, no significant differences between the groups were found with no effect, Mann–Whitney test: IF group *mean rank* = 21.25, PSF group *mean rank* = 20.76, *U* = 205.00, *z* = − 0.13, *p* = 0.45 one-tailed, *d* = 0.04. Hence, hypothesis two is not confirmed.

### Domain-specific self-efficacy

Descriptive data in Table 1 support hypothesis three: Participants self-efficacy in the domain of copyright and media law is higher in the IF group (*M* = 5.30, *SD* = 0.98) than in the PSF group (*M* = 4.66, *SD* = 1.29). This difference is significant with a moderate effect size, Mann–Whitney test: IF group *mean rank* = 24.55, PSF group *mean rank* = 17.62, *U* = 139.00, *z* = − 1.86, *p* < 0.05 one-tailed, *d* = 0.60.

### Cognitive load

Results in Table 1 confirm hypothesis four: For both types of cognitive load participants in the IF group (Overall ICL: *M* = 2.58., *SD* = 1.04; Overall ECL: *M* = 1.94, *SD* = 0.80) report lower values than the PSF group (Overall ICL: *M* = 3.33., *SD* = 1.12; Overall ECL: *M* = 2.29, *SD* = 0.83). For ICL, a Mann–Whitney test reveals that the difference is significant with a moderate effect size: IF group *mean rank* = 17.10, PSF group *mean rank* = 24.71, *U* = 132.00, *z* = − 2.04, *p* < 0.05, *d* = 0.67. However, the difference between the groups regarding the perceived ECL is not significant, the effect size is small, Mann–Whitney test: IF group *mean rank* = 18.48, PSF group *mean rank* = 23.40, *U* = 159.50, *z* = − 1.32, *p* = 0.09 one-tailed, *d* = 0.42.

## Discussion

In this study, we investigated if learning with a digital ERG is more effective when explicit instruction is provided before or after playing the game.

In line with CLT, we predicted in hypothesis one an advantage of the IF design over the PSF design in terms of knowledge retention. Our data support this assumption: learners in the IF condition performed significantly better on the retention test compared to the learners in the PSF condition. We found a moderate effect size with *d* = 0.56 indicating an educational relevant aspect when integrating ERG into teaching. Preparing learners for the problem-based tasks in the ERG through the WBT had a positive effect on mental load. As a result, more cognitive capacities were free during playing the game, enabling a better transfer of new knowledge into long-term memory. This is also reflected in the cognitive load values: The learners in the IF condition reported significantly lower ICL with a moderate effect size (*d* = 67). This means that the participants perceived the instructional design with the WBT followed by the ERG as less complex and difficult resulting in better knowledge achievement. Also, the ECL levels are lower in the IF group with a small but according to Hattie (2008) educational relevant effect size (*d* = 42). However, the difference is not significant, which is probably due to the relatively small sample size. In future studies, researchers should replicate our study with a larger sample to further investigate the possible effect of the instructional design on the ECL. Referring to our data, we can accept hypothesis three as partially confirmed.

In hypothesis two, we assumed according to PF an advantage of the PSF condition in terms of transfer performance. However, we found no differences between the learners engaged in the IF or the PSF instructional design. In both settings, participants performed very well on the transfer task, scoring 16 or more points out of a possible 21. This result is not in line with previous research demonstrating the effectiveness of the PF approach in comparison to direct instructional approaches regarding transfer performance (Kapur, 2011, 2012, 2014). As mentioned earlier, most of the studies reporting advantages of PF over direct instruction were conducted in the field of STEM learning. As shown in Nachtigall et al. (2020), the effectiveness of PF might not occur when applied in non-STEM domains. Learning about copyright and media law is such a non-STEM domain. With this study, we provide further evidence that PF is not better for transfer performance when applied in a non-STEM learning domain compared to an IF instructional design. The claim about the domain-independence of PF effects (Kapur, 2015) therefore needs further investigation. However, we also found no negative effect when the ERG was played before explicit instruction in terms of transfer performance. This is in line with other studies showing that problem-solving first approaches are as effective as more guided instructional approaches with worked-examples or scaffolded guidance before or during instruction (e.g., Halmo et al., 2020). As reported, learners in the PSF condition perceived higher ICL and ECL than the learners in the IF condition. The higher cognitive load only negatively affected learner’s performance on the retention test, not the transfer task. A possible explanation is an affective-motivational effect leading to higher engagement to fulfil the cognitive challenging task (Likourezos & Kalyuga, 2017). Hence, further studies might include measurement of cognitive load (ICL and ECL) and motivational values to further understand the learning process of PSF instructional approaches like the one used here. Since the motivational effect of ERGs and EEGs is also identified as particularly important in the literature (Fotaris & Mastoras, 2019; Veldkamp, van de Grint, et al., 2020), further research on this would be appropriate. It would be exciting to know whether motivation is different in one of the two settings applied in this research. For example, it could be assumed that intrinsic motivation (Ryan & Deci, 2020) is perhaps higher in the instructional design with playing the ERG before instruction due the higher feeling of autonomy generated by the game.

As a third learning outcome in this study, we were interested in learner’s domain-specific self-efficacy after learning with the two respective instructional designs. We predicted that the IF group will report higher self-efficacy compared to the PSF group. Our data confirm this prediction with a moderate effect size (*d* = 0.60): The participants in the IF group felt more self-confident to apply their newly acquired knowledge to solve new problems. Self-efficacy is closely linked to knowledge and therefore in line with the results found regarding the knowledge acquisition. The IF learners performed better on the retention test, and they also felt more self-confident. However, the stronger self-confidence has not resulted in better but the same performance on the transfer task compared to the learners in the PSF condition. Further research is needed to explore if higher self-efficacy translates into better performance on transfer tasks when ERGs are integrated in instructional designs. For example, it is possible that a more difficult transfer tasks would have led to different results. Hence, future research should also vary the instruments evaluating the performance of the learners.

### Limitations and future research

The results observed in this study cannot be generalized due the small sample size. More research is needed to understand when and how to integrate ERGs in educational settings. However, we were able to conduct a randomized experiment with a between-subject design addressing the educational value of an ERG. Such studies were missing as previous research synthesis revealed (Veldkamp, van de Grint, et al., 2020). Another limitation of this study is the individual learning approach that applied as traditionally ERGs are played collaboratively. Follow-up studies are necessary to explore if the same results occur if collaborative learning with the two instructional designs is implemented. Additionally, the instructional designs used in this study should be investigated within physical or hybrid ERGs (like AR escape games; see Paraschivoiu et al., 2021) instead of the fully digital ERG applied in this study. It is also necessary to further investigate instructional elements that might improve learning with EEGs/ERGs. For example, the addition of learning strategies before or after learning with such games could contribute to both retention and transfer performance (Fiorella & Mayer, 2015). Our study addressed a non-STEM learning domain, and therefore, we also encourage researchers to further explore if playing before explicit instruction might be better in STEM domains when compared to playing after explicit instruction.

## Conclusion and implications for practice

In conclusion, the instructional approach of playing an ERG after explicit instruction was more effective to learn about copyright and media law than playing the game before instruction. This is highlighted by the results in the knowledge achievement test, the self-reported domain-specific self-efficacy and the cognitive load levels. Learners in the IF condition showed better knowledge retention, higher self-efficacy and perceived lower cognitive load (ICL and ECL). In addition, the IF learners performed equally on the transfer task. Consequently, we rate the learning experience in the IF group as more effective compared to the learning experience in the PSF group.

Therefore, at this stage of empirical evidence, we recommend teachers, lecturers and instructional designers the implementation of ERGs/EEGs after preparing learners with explicit instruction in the learning domain of the game. As a result, learners will benefit in both knowledge and skills acquisition. In general, we recommend that curriculum designers integrate ERGs/EEGs to create learning opportunities for practicing domain-specific skills. As shown in our study, playing an ERG as part of an instructional design is beneficial when it comes to the application of knowledge. ERGs are, therefore, a new avenue for skill training that is both effective and engaging.

## Supplementary Information

**Additional file 1.** Knowledge acquisition and knowledge application questionnaire used in this study.

## Data Availability

Data can be viewed any time by contacting the first author. The WBT and the ERG are freely available online via wbt.online-lernkurse.de and oer.online-lernkurse.de (in German).

## Abbreviations

CC: Creative Commons
CLT: Cognitive Load Theory
ECL: Extraneous cognitive load
EEG: Educational Escape Game
ERG: Escape Room Game
ICL: Intrinsic cognitive load
IF: Instruction first
OER: Open Educational Resources
PF: Productive Failure
PSF: Problem-solving first
STEM: Science, technology, engineering and mathematics
WBT: Web-based training

## References

[CR1] Bandura A (1977). Self-efficacy: Toward a unifying theory of behavioral change. Psychological Review.

[CR2] Bandura, A. (1994). Self-efficacy. In V. S. Ramachaudran (Ed.), *Encyclopedia of human behavior* (Vol. 4, pp. 71–81). Academic Press. https://www.uky.edu/~eushe2/Bandura/Bandura1994EHB.pdf

[CR3] Borrego C, Fernández C, Blanes I, Robles S (2017). Room escape at class: Escape games activities to facilitate the motivation and learning in computer science. Journal of Technology and Science Education.

[CR4] Cohen, J. (1988). *Statistical power analysis for the behavioural sciences.* (2. Auflage). Academic Press.

[CR5] Cotner, S., Smith, K. M., Simpson, L., Burgess, D. S., & Cain, J. (2018). Incorporating an “Escape Room” game design in infectious diseases instruction. *Open Forum Infectious Diseases*, *5*(suppl_1), S401–S401. 10.1093/ofid/ofy210.1145

[CR6] Duvall, M. (2014). *Adobe Captivate as a Tool to Create eLearning Scenarios*. 514–517. https://www.learntechlib.org/primary/p/148949/

[CR7] Estudante A, Dietrich N (2020). Using augmented reality to stimulate students and diffuse escape game activities to larger audiences. Journal of Chemical Education.

[CR8] Fiorella L, Mayer RE (2015). Learning as a generative activity: Eight learning strategies that promote understanding. Cambridge University Press.

[CR9] Fotaris, P., & Mastoras, T. (2019). Escape rooms for learning: A systematic review. In *Proceedings of the European Conference on Games-Based Learning* (pp. 235–243). 10.34190/GBL.19.179

[CR10] Franco PF, DeLuca DA (2019). Learning through action: Creating and implementing a strategy game to foster innovative thinking in higher education. Simulation & Gaming.

[CR11] Halmo, S. M., Sensibaugh, C. A., Reinhart, P., Stogniy, O., Fiorella, L., & Lemons, P. P. (2020). Advancing the guidance debate: Lessons from educational psychology and implications for biochemistry learning. *CBE—Life Sciences Education*, *19*(3), ar41. 10.1187/cbe.19-11-0260 10.1187/cbe.19-11-0260 PMC8711822 32870078

[CR12] Hattie J (2008). Visible learning: A synthesis over 800 meta-analyses relating to achievement.

[CR13] Hermanns M, Deal B, Campbell AM, Hillhouse S, Opella JB, Faigle C, Campbell RH (2017). Using an “Escape Room” toolbox approach to enhance pharmacology education. Journal of Nursing Education and Practice.

[CR14] Hmelo-Silver CE, Duncan RG, Chinn CA (2007). Scaffolding and achievement in problem-based and inquiry learning: a response to Kirschner, Sweller, and Clark (2006). Educational Psychologist.

[CR15] Huang X (2017). Example-based learning: Effects of different types of examples on student performance, cognitive load and self-efficacy in a statistical learning task. Interactive Learning Environments.

[CR16] Johnson CI, Mayer RE (2009). A Testing effect with multimedia learning. Journal of Educational Psychology.

[CR17] Kapur M (2008). Productive Failure. Cognition and Instruction.

[CR18] Kapur M (2011). A further study of productive failure in mathematical problem solving: Unpacking the design components. Instructional Science.

[CR19] Kapur M (2012). Productive failure in learning the concept of variance. Instructional Science.

[CR20] Kapur M (2014). Productive failure in learning math. Cognitive Science.

[CR21] Kapur, M. (2015). Learning from productive failure. *Learning: Research and Practice*, *1*(1), 51–65. 10.1080/23735082.2015.1002195

[CR22] Kapur M (2016). Examining productive failure, productive success, unproductive failure, and unproductive success in learning. Educational Psychologist.

[CR23] Kerres M, de Witt C (2003). A didactical framework for the design of blended learning arrangements. Journal of Educational Media.

[CR24] Kirschner PA, Sweller J, Clark RE (2006). Why minimal guidance during instruction does not work: An analysis of the failure of constructivist, discovery, problem-based, experiential, and inquiry-based teaching. Educational Psychologist.

[CR25] Klepsch M, Schmitz F, Seufert T (2017). Development and validation of two instruments measuring intrinsic, extraneous, and germane cognitive load. Frontiers in Psychology.

[CR26] Likourezos V, Kalyuga S (2017). Instruction-first and problem-solving-first approaches: Alternative pathways to learning complex tasks. Instructional Science.

[CR27] Loibl K, Roll I, Rummel N (2017). Towards a theory of when and how problem solving followed by instruction supports learning. Educational Psychology Review.

[CR28] Lopez-Pernas S, Gordillo A, Barra E, Quemada J (2019). Analyzing learning effectiveness and students’ perceptions of an educational escape room in a programming course in higher education. IEEE Access.

[CR29] Lopez-Pernas S, Gordillo A, Barra E, Quemada J (2019). Examining the use of an educational escape room for teaching programming in a higher education setting. IEEE Access.

[CR30] Makri A, Vlachopoulos D, Martina RA (2021). Digital Escape rooms as innovative pedagogical tools in education: a systematic literature review. Sustainability.

[CR31] Mayer, R. E. (2019). Computer games in education. *Annual Review of Psychology*, *70*, 531–549. 10.1146/annurev-psych-010418-102744 10.1146/annurev-psych-010418-102744 30231003

[CR32] Mitropoulou, V., & Argyropoulos, N. (2020). *Use of Articulate Storyline 3 to design and develop digital content for an educational platform*. 171–174. https://www.learntechlib.org/primary/p/217299/

[CR33] Nachtigall V, Serova K, Rummel N (2020). When failure fails to be productive: Probing the effectiveness of productive failure for learning beyond STEM domains. Instructional Science.

[CR34] Nicholson, S. (2015). *Peeking behind the locked door: A survey of escape room facilities.* http://scottnicholson.com/pubs/erfacwhite.pdf

[CR35] Nicholson S (2018). Creating engaging escape rooms for the classroom. Childhood Education.

[CR36] Otto, D. (2019). Adoption and diffusion of open educational resources (OER) in education: A meta-analysis of 25 OER-projects. *International Review of Research in Open and Distance Learning*, *20*(5), 122–140. 10.19173/irrodl.v20i5.4472

[CR37] Otto D (2021). Driven by Emotions! The Effect of Attitudes on Intention and Behaviour regarding Open Educational Resources (OER). Journal of Interactive Media in Education.

[CR38] Otto, D., Schroeder, N., Diekmann, D., & Sander, P. (2021). Trends and gaps in empirical research on open educational resources (OER): A systematic mapping of the literature from 2015 to 2019. *Contemporary Educational Technology*, *13*(4), ep325. 10.30935/cedtech/11145

[CR39] Paas F, van Merriënboer JJG (2020). Cognitive-load theory: Methods to manage working memory load in the learning of complex tasks. Current Directions in Psychological Science.

[CR40] Paraschivoiu, I., Buchner, J., Praxmarer, R., & Layer-Wagner, T. (2021). Escape the Fake: Development and Evaluation of an Augmented Reality Escape Room Game for Fighting Fake News. *Extended Abstracts of the 2021 Annual Symposium on Computer-Human Interaction in Play* (pp. 320–325). 10.1145/3450337.3483454

[CR41] Ryan RM, Deci EL (2020). Intrinsic and extrinsic motivation from a self-determination theory perspective: Definitions, theory, practices, and future directions. Contemporary Educational Psychology.

[CR42] Sanchez, E., & Plumettaz-Sieber, M. (2019). Teaching and Learning with Escape Games from Debriefing to Institutionalization of Knowledge. In M. Gentile, M. Allegra, & H. Söbke (Eds.), *Games and Learning Alliance* (Vol. 11385, pp. 242–253). Springer International Publishing. 10.1007/978-3-030-11548-7_23

[CR43] Sinha T, Kapur M (2021). Robust effects of the efficacy of explicit failure-driven scaffolding in problem-solving prior to instruction: A replication and extension. Learning and Instruction.

[CR44] Sweller J (1988). Cognitive load during problem solving: effects on learning. Cognitive Science.

[CR45] Sweller J (2020). Cognitive load theory and educational technology. Educational Technology Research and Development.

[CR46] Sweller J, Kirschner PA, Clark RE (2007). Why minimally guided teaching techniques do not work: A reply to commentaries. Educational Psychologist.

[CR47] Sweller J, Van Merriënboer J, Paas FGWC (1998). Cognitive Architecture and instructional design. Educational Psychology Review.

[CR48] Sweller J, van Merriënboer J, Paas FGWC (2019). Cognitive architecture and instructional design: 20 years later. Educational Psychology Review.

[CR49] Tlili, A., Zhang, J., Papamitsiou, Z., Manske, S., Huang, R., Kinshuk, ... Hoppe, H. U. (2021). Towards utilising emerging technologies to address the challenges of using Open Educational Resources: A vision of the future. *Educational Technology Research and Development,**69*(2), 515–532. 10.1007/s11423-021-09993-4

[CR50] Van Acker F, Van Buuren H, Kreijns K, Vermeulen M, McGreal R, Kinuta W, Marshall S (2013). Why teachers share educational resources: A social exchange perspective. Perspectives on open and distance learning.

[CR51] Veldkamp A, Daemen J, Teekens S, Koelewijn S, Knippels MPJ, Joolingen WR (2020). Escape boxes: Bringing escape room experience into the classroom. British Journal of Educational Technology.

[CR52] Veldkamp A, Knippels M-CPJ, van Joolingen WR (2021). Beyond the Early Adopters: Escape Rooms in Science Education. Frontiers in Education.

[CR53] Veldkamp A, van de Grint L, Knippels M-CPJ, van Joolingen WR (2020). Escape education: A systematic review on escape rooms in education. Educational Research Review.

[CR54] Westera W (2019). Why and How Serious Games can Become Far More Effective: Accommodating Productive Learning Experiences, Learner Motivation and the Monitoring of Learning Gains. Educational Technology & Society.

