# Supplementary material for: Learning with a digital escape room game: before or after instruction?
Source: Res Pract Technol Enhanc Learn. 2022 Mar 15;17(1):10. doi: 10.1186/s41039-022-00187-x (PMC8920572; doi:10.1186/s41039-022-00187-x)
Supplement: Supplementary file 1 — Additional file 1. Knowledge acquisition and knowledge application questionnaire used in this study. [file 41039_2022_187_MOESM1_ESM.docx]

Supplementary Material

Knowledge acquisition questionnaire:

**1. Which material/creations may generally be used freely?** *(1 point)*

☒ Own works

☐ Photos without copyright information

☒ Legal texts

☐ Sheet music

☒ Public domain works

**2. What types of use are permitted under Section 60a UrhG (copyright law)?** *(1 point)*

☒ Duplicate, distribute, make publicly accessible (e.g., intranet / learning platform).

☐ Use in edited form for own works (e.g., scripts)

☐ Commercial use

**3. How many CC licenses does the Creative Commons licensing model include?** *(1 point)*

☐ 4

☒ 6

☐ 9

**4. Which CC license terms represent open educational licenses and are suitable for Open Educational Resources (OER)?** *(1 point)*

☐ CC0

☒ CC BY

☒ CC BY-SA

☐ CC BY-NC

☐ CC BY-ND

**5. What information is required when a work is used and republished under the CC BY 4.0 license?** *(2 points)*

☒ Link to the license text

☒ Author (attribution)

☒ Place of origin

☐ Creation date

**6. Which organization first coined the term OER?** *(2 points)*

☐ European Union (EU)

☐ German Library Association

☒ UNESCO

☐ German Rectors' Conference

Knowledge application test:

**1. May Youtube videos published under the standard Youtube license be downloaded and uploaded as a copy (duplication) in a learning platform (e.g., Moodle / ILIAS)?** *(3 points)*

☒ Only if the video is shorter than 5 minutes or an excerpt that comprises less than 15 percent of the video.

☐ Yes, the video is published on the Internet anyway and therefore freely accessible to everyone.

**2. You would like to accompany a video published under the CC BY license condition with a piece of music under the CC BY-NC license condition and show it in a teaching event with a closed group of participants? Is that allowed?** *(3 points)*

☒ Yes, the two CC licenses for teaching and instruction (non-commercial use) may be combined, provided that all required license information is provided.

☐ No, since the piece of music must be tailored to the length of the video and editing is excluded, the project is not allowed.

☐ No, works with different CC licenses cannot be combined into a new work.

**3. An image is licensed under CC BY-SA 2.0. Which license do you give the image after editing (e.g., cropping)?** *(4 points)*

☐ CC BY 2.0

☒ CC BY-SA 2.0

☐ CC BY-SA 2.1

☐ CC BY-SA-ND 4.0

**4. You decide that a work created by you may be used, edited, and also used for commercial purposes (e.g., advertising) by others. However, you would like to be named as the author. Which license condition do you choose for your work?** *(4 points)*

☐ CC0

☒ CC BY

☐ CC BY-NC

**5. You would like to CC-license your self-created teaching materials. Among the materials are some copyrighted (non-CC-licensed) illustrations that you have scanned from textbooks. Is CC licensing of these illustrators allowed?** *(3 points)*

☐ Yes, that's not a problem at all.

☐ Yes, but I have to credit the source correctly.

☒ No. Content from educational media is generally subject to copyright and may not be digitized or placed under a CC license.

**6. An image is under the CC license CC BY-NC-SA 4.0. how may you use the image?** *(4 points)*

☐ I am not allowed to edit the image.

☒ I may edit the image and use it under the same conditions in my own non-commercial works, provided that I include all necessary license information.

☐ I may offer the image for sale.
